# Type and Extent of Information on (Potentially Critical) Quality Attributes Described in European Public Assessment Reports for Adalimumab Biosimilars

**DOI:** 10.3390/ph14030189

**Published:** 2021-02-25

**Authors:** Ali M. Alsamil, Thijs J. Giezen, Toine C. Egberts, Hubert G. Leufkens, Helga Gardarsdottir

**Affiliations:** 1 Division of Pharmacoepidemiology and Clinical Pharmacology, Utrecht Institute of Pharmaceutical Sciences, Faculty of Science, Utrecht University, 3584 CG Utrecht, The Netherlands; a.m.alsamil@uu.nl (A.M.A.); A.C.G.Egberts@uu.nl (T.C.E.); H.G.M.Leufkens@uu.nl (H.G.L.); 2 Pharmaceutical Product Evaluation Directorate, Drug sector, Saudi Food and Drug Authority, Riyadh 13513-7148, Saudi Arabia; 3 Foundation Pharmacy for Hospitals in Haarlem, 2035 RC Haarlem, The Netherlands; tgiezen@sahz.nl; 4 Department of Clinical Pharmacy, Spaarne Gasthuis, 2035 RC Haarlem, The Netherlands; 5 Department of Clinical Pharmacy, University Medical Center Utrecht, 3584 CX Utrecht, The Netherlands; 6 Department of Pharmaceutical Sciences, University of Iceland, 107 Reykjavik, Iceland

**Keywords:** adalimumab, biosimilar, biosimilarity assessment, quality attributes (QAs), potentially critical quality attributes (pCQAs), European public assessment reports (EPARs)

## Abstract

Regulatory approval of biosimilars predominantly relies on biosimilarity assessments of quality attributes (QAs), particularly the potentially critical QAs (pCQAs) that may affect the clinical profile. However, a limited understanding exists concerning how EU regulators reflect the biosimilarity assessments of (pC)QAs in European public assessment reports (EPARs) by different stakeholders. The type and extent of information on QAs and pCQAs in EPARs were evaluated for seven adalimumab biosimilars. Seventy-seven QAs, including 31 pCQAs, were classified and assessed for type (structural and functional attributes) and extent (biosimilarity interpretation and/or test results) of information in EPARs. Reporting on the QAs (35–75%) varied between EPARs, where the most emphasis was placed on pCQAs (65–87%). Functional attributes (54% QAs and 92% pCQAs) were reported more frequently than structural attributes (8% QAs and 22% pCQAs). About 50% (4 structural and 12 functional attributes) of pCQAs were consistently reported in all EPARs. Regulators often provided biosimilarity interpretation (QAs: 83% structural and 80% functional; pCQAs: 81% structural and 78% functional) but rarely include test results (QAs: 1% structural and 9% functional and pCQAs: 3% structural and 9% functional). Minor differences in structural attributes, commonly in glycoforms and charge variants, were often observed in adalimumab biosimilars but did not affect the functions and clinical profile. Despite the variability in reporting QAs in EPARs, the minor observed differences were largely quantitative and not essentially meaningful for the overall conclusion of biosimilarity of the seven adalimumab biosimilars.

## Highlights

- Comparing adalimumab biosimilars at the level of quality attributes (QAs), as reported in EPARs, showed that the reporting frequencies of QAs vary between biosimilars compared with the same reference biological (Humira^®^).
- Regulators emphasized reporting of potentially critical QAs (pCQAs) in EPARs and more consistently reported functional pCQAs because they are directly related to the drug mechanisms of action and provide valuable information for clinical performance and the extrapolation of indications.
- Regulators often observed minor differences in structural attributes, most commonly in glycoforms and charge variants, between the biosimilar and reference biological, though this had no effect on the functions and clinical profiles and did not preclude biosimilarity.
- Regulators provided a biosimilarity interpretation but rarely reported test results for QAs in EPARs, impeding the interpretation by EPAR users.

## 1. Introduction

Biological drugs have become important treatment options for numerous diseases, including cancer and inflammatory diseases [1]. After patent expiration of the reference biologicals, biosimilars contribute to improved patient access to treatment due to competition, resulting in lower prices. Unlike small molecule drugs, biological drugs, including biosimilars, are large and complicated molecules produced through a complex process using living microorganisms. Variability within and between batches is an inherent feature of the production of biologicals [2,3]. Therefore, biosimilars are, generally, not exact replications of the reference biological but are highly similar [4].

The leading regulatory and health authorities in highly regulated markets, such as the European Medicines Agency (EMA), the United States Food and Drug Administration (US FDA), and the World Health Organization (WHO), have established frameworks and guidelines for the development, assessment, and approval of biosimilars [5,6,7,8]. Biosimilar development and regulatory approval predominantly rely on demonstrating the biosimilarity to the reference biological, which involves a stepwise comparability assessment. The comparability assessment of quality attributes (QAs) is a fundamental step, and it forms the basis for establishing biosimilarity and determining the scope and range of the in-vitro and clinical studies needed for biosimilar approval [9,10,11,12]. Minor differences in QAs between the biosimilar and reference biological may exist but should not be clinically relevant to obtaining regulatory approval.

Quality attributes are measurable molecular characteristics that describe the physical, chemical, biological, and microbiological properties of a drug molecule [13]. Some QAs are classified as potentially critical QAs (pCQAs) because they may affect the biological activity (potency) and the clinical drug profile, which includes pharmacokinetics (PK), pharmacodynamics (PD), safety, immunogenicity, and efficacy [14]. This criticality can be illustrated by a recent example where a biosimilar company discovered a drift in antibody-dependent cell-mediated cytotoxicity (ADCC) activity due to shifts in afucosylated glycans of the reference biological trastuzumab [15], which was associated with a reduced event-free survival rate [16]. Several studies have provided valuable insight into various risk assessment tools for identifying pCQAs [17,18,19,20,21,22]. Some pCQAs apply to all biologicals, but some pCQAs are specific to a biological and information about these may (d)evolve over time as more knowledge of the product and manufacturing process becomes available. The pharmaceutical industry generally defines which QAs are considered pCQAs based on the available information and the manufacturer risk assessment [23,24,25,26,27,28,29,30,31,32]. For biosimilars, the test results of all QAs must remain within the range of variability set by analyzing different batches of the reference biological. Scientific justification is needed if any deviation occurs in the QAs, especially in pCQAs. This rigorous assessment should also be followed when changes are introduced to the manufacturing processes of approved biologicals, including biosimilars [33,34,35,36].

Since the regulatory approval of the first biosimilar in Europe in 2006, 49 unique biosimilars marketed under 69 brand names for 15 reference biologicals have received a positive opinion from the EMA’s Committee for Medicinal Products for Human Use (CHMP) as of November 2020 [37]. Currently, the reference biological adalimumab, sold under the brand name Humira^®^ by AbbVie Corporation, USA, has the largest number of biosimilars approved in the EU market. Adalimumab is an anti-tumor necrosis factor-α (TNF-α) monoclonal antibody that prevents the interaction of TNF-α with its receptors and is indicated for the treatment of various immune-mediated inflammatory diseases [23,38,39].

Despite the established and stringent regulatory pathway of biosimilars in Europe, the adoption of biosimilars in clinical practice is challenged by a lack of knowledge and understanding of the scientific rationale behind their approval [40,41,42]. In Europe, regulators have taken actions to increase transparency for the biosimilar approval process to improve stakeholder understanding of biosimilars through various communication media. The European public assessment report (EPAR) is an unbiased source through which the EMA publishes and broadcasts information to stakeholders about regulatory assessments for all medicinal products approved by the European Commission (EC) [37]. Previous studies have provided an in-depth overview of the clinical evidence reported in EPARs that supports approval of biosimilars in general [43,44] and approval of adalimumab biosimilars in particular [45]. These studies have shown that variations exist in reporting clinical data that confirm the biosimilarity of biosimilars to a reference biological, but they have not explored the reporting of the QAs that are the basis of biosimilar approval. The biosimilarity assessment of QAs is increasingly reported in scientific publications of biosimilars [46], which needed to be systematically consulted with the corresponding EPARs to obtain comprehensive information on biosimilarity at the quality level [47]. However, a limited understanding exists concerning how EU regulators reflect the biosimilarity assessment of (pC)QAs in EPARs by different stakeholders.

Therefore, this study aims to evaluate the QAs and pCQAs reported in EPARs using adalimumab biosimilars as a case study in terms of (1) consistency of QA and pCQA reporting between biosimilars of the same reference biological (i.e., adalimumab), (2) Type of the reported QAs and pCQAs (i.e., structural or functional attributes), and (3) how biosimilarity interpretation and test results were described for the reported (pC)QAs. We hypothesized that EU regulators are more focused in the reporting of pCQAs and the biosimilar interpretation because these are more likely to be of clinical relevance.

## 2. Results

### 2.1. Characteristics of the Included European Public Assessment Reports of Adalimumab Biosimilars

As of 30 November 2020, seven unique adalimumab biosimilars (11 brand names) had received marketing authorization from the EC. Three of the seven biosimilars (i.e., ABP501, GP2017, and MSB11022) were marketed under more than one brand name. Rapporteurs from 11 member states prepared the initial EPARs of the seven adalimumab biosimilars. Rapporteurs from two (Finland and Austria) of the 11 member states were involved in more than one EPAR of adalimumab biosimilars (Table 1).

**Table 1:** Characteristics of the included initial European public assessment reports (EPARs) of adalimumab biosimilars [48,49,50,51,52,53,54,55,56,57,58].

Company Code	Date of Initial EPAR Publication (mm/yyyy)	Brand Names	EU Member State of Rapporteurs (Rapporteur and Co-Rapporteur)
ABP501	04-2017	Amgevita^®^ Solymbic^®^ *	Sweden and Italy
SB5	08-2017	Imraldi^®^	Finland and Austria
BI695501	11-2017	Cyltezo^®^ *	Austria and Germany
GP2017	08-2018	Hefiya^®^ Halimatoz^®^ Hyrimoz^®^	Austria and Ireland
FKB327	09-2018	Hulio^®^	Belgium and United Kingdom
MSB11022	04-2019	Idacio^®^ Kromeya^®^ *	Netherlands and Lithuania
PF06410293	02-2020	Amsparity^®^	Finland and Romania

* Solymbic^®^, Cyltezo^®^ and Kromeya^®^ were approved by the European Medicines Agency (EMA) but voluntarily withdrawn by the applicant for commercial reasons.

### 2.2. Types of Reported (Potentially Critical) Quality Attributes

In general, the frequency of reported QAs (range: 27 (35%)–58 (75%)) varied between EPARs of adalimumab biosimilars, with most emphasis placed on the reporting of the pCQAs (range: 20 (65%)–27 (87%)). The proportion of reported pCQAs was comparable for all biosimilars. Overall, 16 (21%) of all QAs were reported in all EPARs of adalimumab biosimilars. Of the 31 pCQAs, 29 (94%) were reported at least in one EPAR, and 16 (52%) were consistently reported in all included EPARs (Table 2). Two (6%) pCQAs related to structural attributes were not reported in any included EPAR: post-translation modifications (PTMs) including neuraminic N-glycolyl acid and galactose alpha-1,3-galactose (Figure S1).

Overall, functional attributes (54% QAs and 92% pCQAs) were more often consistently reported than structural attributes (8% QAs and 22% pCQAs) in EPARs of adalimumab biosimilars (Table 2). Consistent reporting of functional pCQAs was high, with 12 (92%) out of 13 pCQAs reported in all EPARs, including binding to soluble- and transmembrane-TNFα (s-TNFα and tm-TNFα), (ADCC), and complement-dependent cytotoxicity (CDC) activity and binding to complement component 1q (C1q), neonatal Fc receptor (FcRn), and six Fcγ-receptors. Of the 18 structural pCQAs, only four (22%) were consistently reported in all EPARs, including amino acid sequence and disulfide bridges, glycosylation, and aggregates (Figure S1).

### 2.3. Extent of Information on Reported (Potentially Critical) Quality Attributes

**Figure 1:**
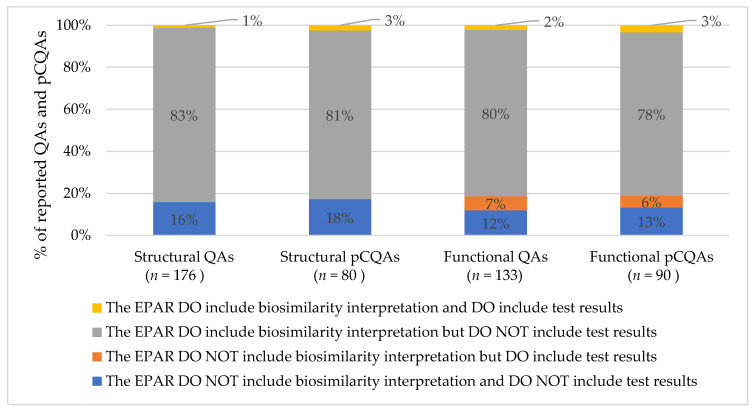
Comparison of the extent of reported information on quality attributes (QAs) and potentially critical quality attributes (pCQAs) stratified by the types of QAs and pCQAs (structural and functional) reported in all EPARs of adalimumab biosimilars included.

In general, no differences were observed in the extent of the reported information between the QAs and pCQAs in all EPARs of adalimumab biosimilars. Regulators frequently provided biosimilarity interpretations of the reported QAs (83% structural and 80% functional) and pCQAs (81% structural and 78% functional) but rarely included test results with or without biosimilarity interpretations of the reported QAs (1% structural and 9% functional) and pCQAs (3% structural and 9% functional) (Figure 1).

The total number of reported QAs included with a biosimilarity interpretation in EPARs was 69 QAs and the number varied (range: 10–58 QAs) for adalimumab biosimilars. The interpretation of the biosimilarity of the reported QAs was most frequently reported as being similar (range: 7–44 QAs) than having minor differences (range: 1–18 QAs) (Table S1). Thirty-one QAs, including fifteen pCQAs, were observed with minor differences in at least one adalimumab biosimilar. The most common structural pCQAs with minor differences were the four glycoforms (galactosylated glycans, high mannose glycans, afucosylated glycans, and sialylated glycans) and the charge variants (acidic and basic variants). While functional pCQAs were more often similar between the biosimilar and reference biological, minor differences were observed for the functional pCQAs tm-TNFα binding, ADCC activity, and C1q binding in two adalimumab biosimilars: GP2017 and PF-06410293 (Figure S1).

Regulators provided both biosimilarity interpretations and test results in EPARs for only five pCQAs, including the protein concentration and binding to FcγRIIIa for ABP501 and the high mannose glycans, ADCC activity, and binding to FcγRIIIa for MSB11022 (Table S2). Of those five pCQAs, only the test results of high mannose glycans, which were slightly lower in the MSB11022 biosimilar (range = 1.9–2.5%) compared to the reference biological (range = 5.3–12.0%), were interpreted by the regulators as minor difference. Figure S1 shows reporting of the type and extent of information on QAs and pCQAs described in the EPARs of adalimumab biosimilars included.

## 3. Discussion

The present study evaluated the type and extent of information on QAs and pCQAs reported in EPARs by EU regulators for seven adalimumab biosimilars approved in Europe as of November 2020. In general, reporting of QAs (ranging from 27 (35%) to 58 (75%)) varied between EPARs of adalimumab biosimilars, where the most emphasis was on reporting pCQAs (ranging from 20 (65%) to 27 (87%)). About 50% (4 structural and 12 functional attributes) of pCQAs were consistently reported in all EPARs. Functional attributes (54% QAs and 92% pCQAs) were more frequently and consistently reported than structural attributes (8% QAs and 22% pCQAs). Minor differences between adalimumab biosimilars and the reference biological in certain structural attributes, most commonly in glycoforms and charge variants, were often observed by regulators. Regulators reported on the biosimilarity interpretation but rarely presented the test results underlying their interpretation in EPARs. However, QA and pCQA data not reported in the EPARs do not necessarily indicate that they were neither submitted by companies nor assessed by regulators during the stringent regulatory process.

This study highlights some variations in reporting biosimilarity assessments at the quality level in EPARs. Despite this variability in QA reporting, pCQAs were most frequently and consistently reported by EU regulators in EPARs. The variation in QA reporting between EPARs is consistent with the variability in reporting clinical data, which was explained by the flexibility in regulatory requirements (i.e., a case-by-case basis) [43,44]. However, such flexibility cannot explain the variability in reporting of QAs and pCQAs for biosimilars, particularly those containing the same active substance and compared to the same reference biological (e.g., Humira^®^ in the case of adalimumab), that were assessed based on the same regulatory standards for establishing biosimilarity. The variability in QA reporting may be explained by the fact that the EPARs are prepared by various rapporteurs (i.e., regulators) from different member states. Nevertheless, regulators diligently reported the pCQAs, which are all considered to be of relevance because these may potentially affect functions (biological and immunochemical activity) and the clinical profile, including the pharmacokinetics, pharmacodynamics, safety, immunogenicity and efficacy of the drug. It is, however, important to note that learning on pCQAs is an ongoing process, which will likely result in changes to the current list over time.

The direct or indirect relationship between structural and functional QAs and the clinical profile influences the determination of pCQAs [19]. This relationship can be illustrated by the four structural pCQAs, including the amino acid sequence, disulfide bridges, aggregates, and glycosylation, which were consistently reported in EPARs. A mismatch in amino acid sequence and disulfide bridges can change the structural conformation affecting the biological activity and clinical performance, which were identical to the reference biological for all adalimumab biosimilars. Aggregates can elicit immunogenic responses by inducing neutralizing antibodies, hypersensitivity reactions, and infusion-related reactions in vivo. The propensity of aggregation may increase with some structural attributes (e.g., disulfide bridges, oxidation, and deamidation) if these are inadequately controlled. For all adalimumab biosimilars, aggregate levels were similar to the reference biological. Glycosylation is a PTM that occurs through an enzymatic process at specific sites in a protein drug and can influence the biological activity (potency and efficacy), serum half-life clearance (pharmacokinetics), and immunogenicity (safety). Minor differences in glycosylation were observed in adalimumab biosimilars, which are the most frequent notable differences in biosimilars and reference biologicals in general [9,10,11,12].

In practice, minor differences in QAs and pCQAs are expected for biosimilars due to the use of various manufacturing processes, cell lines, and materials [35]. These minor differences have also been observed between batches of a reference biological, primarily when a company introduces manufacturing changes [2,3,23]. The galactosylated glycans, high mannose glycans, afucosylated glycans, and sialylated glycans are types of glycoforms where minor differences have most commonly been reported (Figure S1). Galactosylated glycans may influence C1q binding and CDC activity, whereas high mannose glycans may influence pharmacokinetics parameters. However, structure-activity relationship studies and pivotal pharmacokinetics trials indicate that these are not affected by minor differences in galactosylated and high mannose glycans [48,49,51,52,53,54,55,56,57]. The same applies to afucosylated and sialylated glycans, which may influence Fcγ-receptors and ADCC activity [51,52,53,54,55,56,57,58]. These examples demonstrate the importance of structure-activity relationship studies and pharmacokinetics and pharmacodynamics trials in assessing the potential effect of minor differences in pCQAs in biosimilarity assessments. Minor differences in acidic and basic variants in several adalimumab biosimilars were attributed to changes in c-terminal lysin [48,49,51,52,53,54,58], which is generally cleaved in human serum with no effect on clinical profiles, and were thus considered noncritical QAs. Minor differences for certain functional pCQAs were attributed to minor differences in certain structural QAs and pCQAs, which were observed and reported by EU regulators in EPARs for GP2017 and PF06410293. For both biosimilars, the minor differences in ADCC activity disappeared when using an in-vitro assay with more physiological conditions in peripheral blood mononuclear cells. For GP2017, the aggregate levels were slightly higher using size-exclusion chromatography and slightly lower using analytical ultracentrifugation than the reference biological, which was considered a minor and clinically irrelevant difference by regulators. This ADCC and aggregate example indicates the importance of using orthogonal methods to assess the (dis)similarity of QAs. Based on these observations, minor differences in these pCQAs seem to be quantitative (i.e., numerical values) but do not preclude the overall conclusion for biosimilarity and are considered clinically irrelevant.

The underlying reason functional pCQAs are more frequently and consistently reported in EPARs could relate to their direct relationship with the mechanisms of action (MoAs). The primary MoA of adalimumab involves binding to, and neutralizing TNF-α. Adalimumab also mediates effector functions, such as ADCC and CDC activity, by binding to tm-TNF-α, C1q (for CDC), and Fcγ-receptors. The relevance of ADCC or CDC activity to the primary MoA and efficacy of adalimumab is not well established but may be important, particularly in inflammatory bowel disease [45]. Binding to tm-TNFα can trigger potential biological functions known as “referred signaling,” which may play a role in some therapeutic indications (e.g., inflammatory bowel disease). For GP2017, regulators reported minor differences in the binding to tm-TNFα, for which the scientific justifications provided by the company were not available in the EPAR for GP2017. However, the developer company of GP2017 reported functional and pharmacological characterizations demonstrating indistinguishable binding profiles and subsequent induction of reverse signaling to support the rationale for extrapolation across indications [28]. Therefore, functional pCQAs provide the final insight into the (dis)similarity at the quality level and useful information in predicting the outcomes of clinical studies [9,10,11], forming the basis for supporting the extrapolation of biosimilars across all indications authorized for the reference biological [59,60,61,62].

Regulators frequently describe the biosimilarity interpretation of reported QAs and pCQAs but rarely present the test result data, impeding the interpretation by EPAR users. For example, in EPARs, minor differences are frequently expressed subjectively as “slightly lower” or “slightly higher,” but the exact extent to which the difference is minor remains unclear for most reported QAs and pCQAs. A more appropriate method would be in line with what was reported in the EPAR of MSB11022, in which the ranges of high mannose glycans (ranging from 1.9% to 2.5%) and the reference adalimumab (ranging from 5.3% to 12.0%) were reported. Such information on the test results allows for a better understanding of the regulatory interpretation and scientific justification behind the regulatory approval of biosimilars.

The present study used a classification scheme to investigate in a standardized manner how EU regulators present information on the biosimilarity of QAs and pCQAs in EPARs. The focus on the pCQAs to be considered in biosimilarity assessment, which may affect the clinical profiles of adalimumab products, was a strength of this investigation. The selection of adalimumab pCQAs was based on the literature review, providing an overview concerning which QAs are considered pCQAs with the current knowledge. This study stresses the importance of EPARs as a source of information that provides insight into the scientific evidence underpinning the regulatory approval of biosimilars.

Our study does have some limitations, which are noted as follows. First, these study findings are restricted to adalimumab biosimilars, which may hamper the generalizability to biosimilars of other biological molecules. Nevertheless, even if a biosimilarity assessment of another molecule is conducted with a different set of QAs and pCQAs, the findings, especially the focus on reporting the pCQAs, are expected to be comparable to other types of biosimilars because all EPARs are published by the same regulatory agency (i.e., EMA). Second, the generalizability of our findings to the regulatory reports from various jurisdictions, such as in the US FDA review reports, is unknown and beyond the scope of this study. Third, the QA classification scheme may not have captured all pCQAs of adalimumab because no consensus list is currently available. However, a literature search for publications on comparability and biosimilarity studies of adalimumab products was performed, and no pCQAs were identified that were not included in our classification.

Our observations reveal that minor differences in certain QAs between biosimilars and reference biological can occur at the same level of variability between pre- and post- manufacturing change batches of the reference biological [23,35,63], which reassures the biosimilar regulation system. Although EU regulators have focused on describing pCQAs, these critical attributes were not explicitly defined in EPARs. Because biosimilar companies have conducted extensive analyses to define pCQAs based on their risk assessments, it would be preferable if regulators clearly define which QAs are identified as pCQAs by the companies. A clear definition of pCQAs in EPARs would enable stakeholders to better understand the links between QAs and the clinical profile and the meaning of the QAs concerning patient safety and product efficacy. The pCQAs may also (d)evolve over the drug life cycle based on the knowledge gained regarding the product and process. Standardized reporting of pCQAs in EPARs would benefit regulatory learning by allowing future researches to track pCQAs over time. Learning of pCQAs over time might result in reducing the need for comparative clinical trials and streamlining biosimilar approvals [9,10,11,12].

Although the EMA quality guidance of biosimilars provides high-level information on QAs, the guidance was last updated in 2014 and may not reflect the current state of knowledge and regulatory experience regarding QAs for biosimilars [5]. The lack of information on pCQAs in the guidance is understandable because these were not entirely known in the early years of biosimilar regulation. Nevertheless, the accumulated and long experience of EU biosimilar regulation as reflected in EPARs would fuel regulatory guidance with product-specific pCQAs, making the regulatory standard more visible and predictable.

As EPARs are considered an unbiased information source, there is great value in providing insight into the biosimilarity assessment of QAs for various stakeholders involved in biosimilar development, adoption, and regulation. The pharmaceutical industry can use EPARs to learn from past successes and failures and predict the regulatory process, and EPARs as such may contribute to reducing the time and cost of biosimilar development [64]. Healthcare professionals (HCPs) can use EPARs to understand the QA assessment’s crucial role during the regulatory approval of biosimilars [65,66]. Reporting more extensive information about pCQAs in EPARs could help HCPs understand the predominant role of QAs and the reduced weight of evidence from comparative clinical trials in biosimilar approval. Among HCPs, pharmacists are uniquely positioned to take a leading role in informing other HCPs and patients about the scientific evidence underpinning biosimilar approval. Such efforts could increase confidence in and acceptance of using biosimilars in medical practice to fully capture the societal and patients benefits offered by biosimilars. Non-European regulatory authorities can use EPARs to support their own decision-making process, relying on the regulatory assessment undertaken by competent authorities in the world [67,68,69,70,71,72]. Therefore, EPARs could contribute to accelerating the regulatory review process and patients access to biosimilars in non-European jurisdictions.

For a comprehensive understanding of biosimilarity concepts and the predominant role of QAs in the approval of biosimilars, continued improvement in presenting biosimilarity assessments of QAs in EPARs is recommended. One method could include applying a structured uniform approach to QA reporting in EPARs. Such an approach may enhance the completeness and consistency of QA data and avoid missing crucial regulatory reflection on clinically relevant pCQAs. Greater consistency in QA reporting could make the EPAR a valuable and reliable tool for stakeholders to support evidence-based education to address the lack of knowledge and understanding of the scientific rationale behind biosimilar approval. Biosimilarity assessments of QAs in EPARs could be summarized in a standardized format that includes the type of evaluated QAs, explicit definition of the pCQAs, the test methods used and their results, the biosimilarity interpretation and scientific justification of the differences, if applicable. This summary could be achieved through adopting the International Pharmaceutical Regulators Program’s regulatory review templates to optimize the current content with respect to biosimilarity assessment of QAs in EPARs [73]. Alternatively, initiating a project similar to the collaborative study between the EMA and European network for health technology assessment [74], which has resulted in a template to improve the contribution of EPARs in health technology assessments of relative drug effectiveness.

## 4. Methods

### 4.1. Study Cohort

In this study, the initial EPARs of all adalimumab biosimilars approved by the EMA before 30 November 2020 were included. The initial EPARs of adalimumab biosimilars were retrieved from the official EMA website (http://www.ema.europa.eu (accessed on 1 June 2020 )) [37]. The EPAR contains a summary of the submitted registration dossier and the scientific assessment undertaken by the CHMP, a body that advises the EC on marketing authorization of medicines for human use. Only the initial EPAR of each adalimumab biosimilar released following the final EC decision was included in this study because biosimilarity assessments of QAs and pCQAs between biosimilar and reference biological are presented only in the initial EPARs.

The initial EPARs were used to extract baseline characteristics for each adalimumab biosimilar, including the company code(s), brand name(s), date of the initial EPAR publication, and member states of the rapporteurs responsible for the assessment. Some adalimumab biosimilars are produced by the same manufacturer but marketed under different brand names (e.g., the company code for Hefiya^®^, Halimatoz^®^, and Hyrimoz^®^ is GP2017) for which the registration dossier and initial EPARs are identical. In such cases, only the EPAR of one brand name (e.g., Hefiya^®^ for GP2017) was included in the study for subsequent analysis. The date of the initial EPAR publication was defined as the month and year when the EPAR was published by the EMA, which is generally the same date as the EC decision on marketing authorization. The member state was defined as the rapporteurs’ European country of origin. The rapporteurs are the two CHMP members who led the regulatory assessment of a marketing authorization application.

### 4.2. Information on (Potentially Critical) Quality Attributes in EPARs

The study outcome was the determination of how EU regulators report information on the biosimilarity assessment of QAs and pCQAs in the EPARs. Two aspects were studied: the type and extent of information on the reported QAs and pCQAs.

#### 4.2.1. Types of Reported (Potentially Critical) Quality Attributes

**Figure 2:**
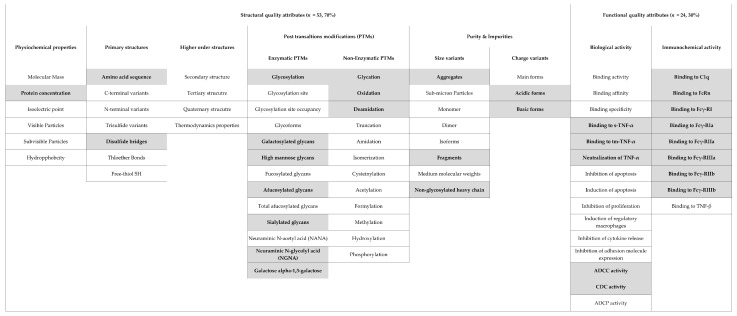
Classification scheme for 77 common quality attributes (QAs) of biologicals including 31 potentially critical quality attributes (pCQAs) relevant to adalimumab. The pCQAs are presented in gray boxes. Definitions: ADCC: antibody-dependent cellular cytotoxicity, ADCP: antibody-dependent cellular phagocytosis, CDC: complement-dependent cytotoxicity, C1q: complement component 1q, TNFα: tumor necrosis factor-alpha, s-TNFα: surface tumor necrosis factor-alpha, tm-TNFα: transmembrane tumor necrosis factor-alpha, Fc: fragment crystallizable, FcR: Fc receptor.

The types of QAs and pCQAs reported in the biosimilarity assessment were identified from the quality, non-clinical, and clinical sections of the initial EPARs. A general classification scheme of QAs was used to extract information from the EPARs. Information about the development of the classification scheme has been described elsewhere [46]. In short, the first draft was developed by the authors based on information from the EMA and US FDA biosimilar guidelines [5,6,7] and publicly available information relevant to the molecular characterization of a biological drug. The classification scheme was validated by regulators involved in the quality assessment of biosimilars at the Dutch Medicines Evaluation Board (MEB) to ensure that no critical and relevant QAs were missed. The classification scheme divides the QAs into seven types with additional subtypes of structural (physiochemical properties, primary structure, higher-order structure, PTMs and purity and impurities) and functional attributes (biological and immunochemical activity), resulting in the classification of 77 (53 structural and 24 functional) QAs of biologicals considered in the biosimilarity assessment (Figure 2) [46,47].

Subsequently, a list of pCQAs was defined in a two-step process. First, the pCQAs of adalimumab were identified from scientific publications presenting comparability or biosimilarity studies of adalimumab products, including the reference biological (Humira^®^) and corresponding biosimilars [23,24,25,26,27,28,29,30,31,32]. The publications were selected from an updated search of our previous systematic review [46]. From this search, an initial list of 29 pCQAs of adalimumab was constructed based on the pCQAs proposed by the authors. Second, the initial list was compared with the pCQAs identified for monoclonal antibodies, in general, in the previous literature [17,18,19,20,21,22] to verify and broaden the initial selection of pCQAs. If a new pCQA was identified in this second step, the authors (A.M.A., T.J.G., and H.G.) discussed its relevancy to adalimumab and reached a consensus on the inclusion of the attribute. In this way, two pCQAs were added to the initial list, resulting in a final list of 31 (18 structural and 13 functional) pCQAs considered relevant to adalimumab products. These pCQAs were classified according to the previously described scheme (Figure 2).

#### 4.2.2. Extent of Reported Information on (Potentially Critical) Quality Attributes

The extent of the information on QAs and pCQAs provided in the EPARs was categorized by whether a biosimilarity interpretation was reported (yes/no) and whether test results were reported (yes/no) for a given QA or pCQA. The four possible combinations of answers resulted in four categories for each reported QA and pCQA (Table 3) [47].

Biosimilarity interpretation was defined as reported (yes) if the EPAR contained keywords demarcating the regulatory interpretation of the biosimilarity of a QA and pCQA as identical, similar, or having minor differences. The interpretation of *similar* included wording such as “same,” “match,” “(highly) similar,” “comparable,” and “consistent”.

Test results were defined as reported (yes) if the EPAR included the quantitative or qualitative acceptance criteria of a given QA and pCQA, which included the numerical limits, range, and distribution, as shown in the examples in Table 3, or other suitable visual assessment measures, such as the spectra for higher-order structures and chromatograms for purity and impurities.

### 4.3. Data Analysis

The frequency of the reported QAs and pCQAs stratified by structural and functional attributes was used to express the consistency in reporting the QAs and pCQAs of adalimumab biosimilars by EU regulators in EPARs. A QA and pCQA was considered to be consistently reported if EU regulators describe it in all included EPARs. The proportion of reported QAs and pCQAs for the four reporting categories (see Table 3) was calculated and stratified by structural and functional attributes to compare the extent of information on reported QAs and pCQAs in EPARs. If the regulatory interpretation of the biosimilarity or test results were presented for a given QA or pCQA in the EPARs, the type of interpretation (identical, similar, or minor differences) and the acceptance biosimilarity criteria were identified.

## 5. Conclusions

In conclusion, we found variations in the frequency of reported QAs between EPARs of adalimumab biosimilars. The minor differences in the identified QAs did not affect functions and clinical performance and seem to be largely quantitative differences and not essentially meaningful for the overall conclusion of biosimilarity.

In line with our hypothesis, the pCQAs, specifically functional pCQAs, were reported most frequently and consistently in EPARs, as these reflect the MoA and can potentially affect the clinical profile. Greater consistency could be applied in reporting of QAs with more emphasis on pCQAs in EPARs, which could improve the understanding of the relationship between QAs and the clinical profile, which may positively contribute to adopting biosimilars in clinical practice.

## Figures and Tables

**Table 2:** Reporting of the quality attributes (QAs) and potentially critical quality attributes (pCQAs) stratified by structural and functional attributes and the company code of adalimumab biosimilars in the included European public assessment reports (EPARs).

Company Code	All QAs (*n* = 77, 100%)	Type of QAs		All pCQAs (*n* = 31, 100%)	Type of pCQAs	
		Structural (*n* = 53, %)	Functional (*n* = 24, %)		Structural (*n* = 18, %)	Functional (*n* = 13, %)
ABP501	36 (47%)	18 (34%)	18 (75%)	20 (65%)	7 (39%)	13 (100%)
SB5	49 (64%)	27 (51%)	22 (92%)	27 (87%)	14 (78%)	13 (100%)
BI695501	27 (35%)	12 (23%)	15 (63%)	20 (65%)	7 (39%)	13 (100%)
GP2017	52 (68%)	34 (64%)	18 (75%)	27 (87%)	14 (78%)	13 (100%)
FKB327	58 (75%)	39 (74%)	19 (79%)	27 (87%)	14 (78%)	13 (100%)
MSB11022	42 (55%)	20 (38%)	22 (92%)	25 (81%)	12 (67%)	13 (100%)
PF06410293	46 (60%)	27 (51%)	19 (79%)	24 (77%)	12 (67%)	12 (92%)
Consistent for all biosimilars	16 (21%)	4 (8%)	12 (54%)	16 (52%)	4 (22%)	12 (92%)

**Table 3:** Definitions of the four reporting categories for the quality attributes (QAs) and potentially critical quality attributes (pCQAs) reported in biosimilarity assessments in the initial European public assessment reports (EPARs) [47].

Reporting Catagories	Biosimilarity Interpretation		
	No		Yes
Test results	No	Reported QAs and pCQAs include no biosimilarity interpretation and no test results, for example: - The amino acid sequence and *N*-glycosylation site were compared. - The protein concentration was determined. - Binding to FcRn and Fcγ-RIIIa was studied, and a comparison of ADCC activity was performed. - Neutralization of TNFα, binding to s-TNFα, and binding to tm-TNFα were addressed.	Reported QAs and pCQAs include the biosimilarity interpretation but not test results, for example: - The amino acid sequence and *N*-glycosylation site of the biosimilar were identical to those of the reference. - The protein concentration was similar to that of the reference. - Minor differences with no clinical relevance were observed in glycation, galactosylated *N*-glycans, high mannose *N*-glycans, fucosylated N-glycans, and sialylated glycans. - The FcRn, C1q binding, CDC, ADCC, and neutralization of TNFα were comparable with those of the reference.
	Yes	Reported QAs and pCQAs include the test results but not the biosimilarity interpretation, for example: - The levels of high mannose *N*-glycans (biosimilar: 1.9–2.5%; reference: 5.3–12.0%). - The K_D_ ranges for Fcγ-RIIIa binding (biosimilar: 6.2–10.1 nM; reference: 3.8–8.0 nM) - The EC_50_ values for inhibiting cytokine release (204 pM, 294 pM and 200 pM for the three batches of tested biosimilars and 177 pM, 168 pM and 222 pM for the three batches of tested reference biological). - ADCC activity (biosimilar: 89–107%; reference: 84–115%)	Reported QAs and pCQAs include the biosimilarity interpretation and test results, for example, - Minor differences with no clinical relevance were observed in the levels of high mannose *N*-glycans (biosimilar: 1.9–2.5%; reference: 5.3–12.0%). - ADCC activity (biosimilar: 89–107%; reference: 84–115%) was comparable/similar between the two products.

ADCC: antibody-dependent cellular cytotoxicity, CDC: complement-dependent cytotoxicity, EC_50_: half-maximal effective concentration, TNFα: tumor necrosis factor-alpha, s-TNFα: surface tumor necrosis factor-alpha, tm-TNFα: transmembrane tumor necrosis factor-alpha, Fc: fragment crystallizable, FcR: Fc receptor, K_D_: equilibrium dissociation constant, nM: nanomoles, pM: picomoles.

## Data Availability

The datasets during and/or analyzed during the current study are available from the corresponding author on reasonable request.

## Supplementary Materials

The following are available online at https://www.mdpi.com/1424-8247/14/3/189/s1, Figure S1: The types of and extent of information on quality attributes (QAs) and potentially critical QAs (pCQAs, in bold and gray boxes) as part of biosimilarity assessment reported by regulators in the initial European public assessment reports (EPARs) of seven adalimumab biosimilars; Table S1: Types of biosimilarity interpretation of reported quality attributes (QAs) stratified by the company code of adalimumab biosimilars in the European public assessment reports (EPARs); Table S2: Comparison of potentially critical quality attributes (pCQAs) where test results and interpretation were reported for ABP501 and MSB11022 biosimilar.
