# Supplementary material for: Type and Extent of Information on (Potentially Critical) Quality Attributes Described in European Public Assessment Reports for Adalimumab Biosimilars
Source: Pharmaceuticals (Basel). 2021 Feb 25;14(3):189. doi: 10.3390/ph14030189 (PMC7996580; doi:10.3390/ph14030189)
Supplement: Supplementary file 1 [file pharmaceuticals-14-00189-s001.zip › pharmaceuticals-1080544-supplementary.pdf]

Supplementary materials

Category		Quality Attributes (QAs)		ABP501	SB5	BI65501	GP2017	FKB327	MSB11022	PF06410293
Physiochemical properties		QA1	Molecular Mass	○	◐≠	○	◐=	◐=		◐=
		**QA2**	**Protein concentration***	●=	◐=		◐=	◐=	◐=	◐=
		QA3	Isoelectric point				◐=	◐=		
		QA4	Visible Particles	◐=		◐≠	◐=	◐=		
		QA5	Subvisible Particles	◐=	◐=		◐=	◐=		
		QA6	Hydrophobicity				◐≠	◐≠		
Primary structure		**QA7**	**Amino acid sequence***	◐=	○	○	◐=	◐=	◐=	◐=
		QA8	C-terminal variants	◐≠	◐≠		◐≠	◐≠	◐=	
		QA9	N-terminal variants		○		◐≠	◐=	◐=	
		QA10	Trisulfide variants					◐≠		
		**QA11**	**Disulfide bridges***	○	○	○	◐=	◐=	◐=	◐=
		QA12	Thioether Bonds				◐≠	◐=		
		QA13	Free-thiol SH		◐≠		◐=	◐=	◐=	◐=
Higher order structures		QA14	Secondary structure	○	◐=	○	◐=	◐=		◐=
		QA15	Tertiary structure	○	◐=		◐=	◐=		◐=
		QA16	Quaternary structure				◐=			
		QA17	Thermodynamics properties	○			◐=	◐=		◐=
Post-Translation Modifications-PTMs	Enzymatic- PTMs	**QA18**	**Glycosylation***	◐=	◐≠	○	◐≠	◐=	◐=	◐=
		QA19	Glycosylation site	○	◐=	○		◐=		◐=
		QA20	Glycosylation site occupancy		○		◐≠	◐=		◐=
		QA21	Glycoforms	◐≠	◐≠		◐≠	◐≠	◐≠	◐≠
		**QA22**	**Galactosylated glycans***	◐≠	○		◐≠	◐≠	◐=	◐≠
		**QA23**	**High mannose glycans***	◐≠	○		◐≠	◐≠	●≠	◐≠
		QA24	Fucosylated glycans		◐≠			◐≠		
		**QA25**	**Afucosylated glycans***		◐≠		◐≠	◐≠		◐≠
		QA26	Total afucosylated glycans						◐≠	◐≠
		**QA27**	**Sialylated glycans***		◐≠			◐≠	◐≠	◐=
		QA28	Neuraminic N-acetyl acid (NANA)							◐=
		**QA29**	**Neuraminic N-glycolyl acid (NGNA)***							
		**QA30**	**Galactose alpha-1,3-galactose***							
	Non-Enzymatic- PTMs	**QA31**	**Glycation***				◐≠	◐=	◐=	
		**QA32**	**Oxidation***		◐≠	○	◐=	◐=	◐=	
		**QA33**	**Deamidation***		○	○	◐=	◐=	◐=	
		QA34	Truncation	○	○	○	◐=	◐≠		
		QA35	Amidation				◐=	◐≠		
		QA36	Isomerization				◐=	◐=		
		QA37	Cysteinylation					◐=		
		QA38	Acetylation							
		QA39	Formylation							
		QA40	Methylation							
		QA41	Hydroxylation							
		QA42	Phosphorylation							
Purity and impurities	Size variants	**QA43**	**Aggregates***	○	◐=	○	◐≠	◐=	◐=	◐=
		QA44	Sub-micron Particles	○						
		QA45	Monomer		◐≠		◐=	◐=	◐=	◐=
		QA46	Dimer				◐≠		◐=	◐=
		QA47	Isoforms						◐=	◐=
		**QA48**	**Fragments***			○	◐=	◐=	◐=	◐=
		QA49	MMWs					◐=		
		**QA50**	**Non-glycosylated heavy chain (NGHC)***		◐≠					
	Charge variants	QA51	Main forms					◐≠		◐=
		**QA52**	**Acidic forms***		◐≠		◐≠	◐≠		◐=
		**QA53**	**Basic forms***		◐≠		◐≠	◐≠		◐=
Biological activity		QA54	Binding activity		○		◐=	◐=	◐=	◐=
		QA55	Binding affinity		◐=		◐=	◐=	◐=	◐=
		QA56	Binding specificity	○	○		◐=	◐=	◐=	◐=
		**QA57**	**Binding to s-TNFα***	◑	◐=	○	◐=	◐=	◐=	◐=
		**QA58**	**Binding to tm-TNFα***	◑	◐=	○	◐≠	◐=	◐=	◐=
		**QA59**	**Neutralization of TNFα***	◑	◐=	○	◐=	◐=	◐=	
		QA60	Inhibition of apoptosis	◑	◐=		◐=	◐=	◐=	◐=
		QA61	Induction of apoptosis	◑	◐=	◐=			◐=	
		QA62	Inhibition of proliferation	◑				◐=	◐=	◐=
		QA63	Induction of regulatory macrophages		◐=			◐=	◐=	◐=
		QA64	Inhibition of cytokine release	◑	◐=		○		◐=	
		QA65	Inhibition of adhesion molecule expression		◐=				◐=	◐=
		**QA66**	**ADCC activity***	○	◐=	○	◐≠	◐=	●=	◐≠
		**QA67**	**CDC activity***	○	◐=	◐=	◐=	◐=	◐=	◐=
		QA68	ADCP activity			◐=				
Immunochemical activity		**QA69**	**Binding to C1q***	○	◐=	○	◐≠	◐=	◐=	◐=
		**QA70**	**Binding to FcRn***	◐=	◐=	○	◐=	◐=	◐=	◐=
		**QA71**	**Binding to Fcγ-RI***	○	◐=	◐=	◐=	◐=	◐=	◐=
		**QA72**	**Binding to Fcγ-Ria***	◑	◐=	◐=	◐=	◐=	◐=	◐=
		**QA73**	**Binding to Fcγ-RIIa***	◑	◐=	◐=	◐=	◐=	◐=	◐=
		**QA74**	**Binding to Fcγ-RIIIa***	●=	◐=	◐=	◐=	◐=	●=	◐=
		**QA75**	**Binding to Fcγ-RIIb***	○	◐=	◐=	◐=	◐=	◐=	◐=
		**QA76**	**Binding to Fcγ-RIIIb***	○	◐=	◐=	◐=	◐=	◐=	◐=
		QA77	Binding to TNF-β		◐=					◐=

Supplementary Figure S1: The types of and extent of information on quality attributes (QAs) and potentially critical QAs (pCQAs, in bold and gray boxes) as part of biosimilarity assessment reported by regulators in the initial European public assessment reports (EPARs) of seven adalimumab biosimilars

- ○ QAs reported in the EPAR DO NOT include biosmilarity interpretation and DO NOT include test results
- ◑ QAs reported in the EPAR DO NOT include biosmilarity interpretation and DO include test results
- ◐ QAs reported in the EPAR DO include biosmilarity interpretation and DO NOT include test results
- ● QAs reported in the EPAR DO include biosmilarity interpretation and DO include test results and
- = Biosimilarity interpretation of the test result of QA was similar between biosimilar and reference biological
- ≠ biosmilarity interpretation of the test result of QA was different between biosimilar and reference biological

Supplementary Table S1: Types of biosimilarity interpretation of reported quality attributes (QAs) stratified by the company code of adalimumab biosimilars in the European public assessment reports (EPARs)

Company code	All QAs (n=77; 100%)	QAs reported with biosimilarity interpretation (n)	Type of biosimilarity interpretation			
			Similar		Minor differences	
			Structural QAs (n)	Functional QAs (n)	Structural QAs (n)	Functional QAs (n)
ABP501	36 (47%)	11	5	2	4	0
SB5	49 (64%)	39	6	20	13	0
BI695501	27 (35%)	10	0	9	1	0
GP2017	52 (68%)	51	19	14	15	3
FKB327	58 (75%)	58	25	19	14	0
MSB11022	42 (55%)	42	16	22	4	0
PF06410293	46 (60%)	46	21	19	5	1

Supplementary Table-S2: Comparison of potentially critical quality attributes (pCQAs) where test results and interpretation were reported for ABP501 and MSB11022 biosimilar

**Biosimilar**	**Quality attributes**	**Test result**	**Biosimilarity interpretation**
ABP501	**Protein concentration (mg/ml)**	ABP501 [range (n)]:<br>50.2 – 52.6 (4)<br><br>US Reference [range (n)]:<br>51.1– 53.1 (3)<br><br>EU Reference [range (n)]:<br>50.6 – 51.6 (3)	Similar
	**Binding to FcgRIIIa (%)**	ABP501 [mean (SD)]:<br>108 (12.3)<br><br>US Reference [mean (SD)]:<br>101 (13.6)<br><br>EU Reference [mean (SD)]:<br>113 (7.6)	Similar
MSB11022	**High mannose glycans (%)**	MSB11022 [range (n)]:<br>1.9-2.5 (NR)<br><br>EU Reference [range (n)]:<br>5.3-12.0 (NR)	Minor differences
	**Binding to FcgRIIIa (nM)**	MSB11022 [range (n)]:<br>6.2 – 10.1 (NR)<br><br>EU Reference [range (n)]:<br>3.8 – 8.0 (NR)	Similar
	**ADCC activity (%)**	MSB11022 [E_{max} range(n)]:<br>84-92 at F/F genotype<br>88-99 at V/F genotype<br><br>MSB11022 [EC_{50} range(n)]:<br>41-56 at F/F genotype<br>26-37 at V/F genotype<br><br>EU Reference [E_{max} range(n)]:<br>95-104 at F/F genotype<br>94-104 at V/F genotype<br><br>EU Reference [E_{max} range(n)]:<br>79-162 at F/F genotype<br>70-174 at V/F genotype	Similar

ADCC, Antibody Dependent Cellular Cytotoxicity, EC_{50}, half-maximal effective concentration, E_{max}, maximal effect at high drug concentrations, EU, European Union, US, United States. NR, not reported; FcgRIIIa, Fragment crystallizable gamma receptor; mg/ml; milligram/ milliter; nM, nanomole
